# Effect of UV-C Radiation on Genomic Variation in *Chlamydomonas reinhardtii*

**DOI:** 10.3390/genes17050563

**Published:** 2026-05-13

**Authors:** Rosa Paola Radice, Francesca Padula, Valeria Iannelli, Xavier Montagnuolo, Antonio Scopa, Marios Drosos, Giuseppe Martelli

**Affiliations:** 1 Department of Base and Applied Science, University of Basilicata, Viale dell’Ateneo Lucano 10, 85100 Potenza, Italy; francesca.padula@unibas.it (F.P.); giuseppe.martelli@unibas.it (G.M.); 2 Department of Agriculture, Forest, Food, and Environmental Sciences, University of Basilicata, Viale dell’Ateneo Lucano 10, 85100 Potenza, Italy; valeria.iannelli@unibas.it (V.I.); antonio.scopa@unibas.it (A.S.); marios.drosos@unibas.it (M.D.); 3 AlgaebioMed Srl, via Luigi Kossuth 7, 00149 Roma, Italy

**Keywords:** UV-C radiation, *Chlamydomonas reinhardtii*, random mutagenesis, genomic variability, oxidative stress, DNA repair, microalgae, gene expression, photosynthesis, strain improvement

## Abstract

Background: Ultraviolet-C (UV-C) radiation is a high-energy physical mutagen capable of inducing DNA damage and oxidative stress, thereby generating genomic variability in photosynthetic organisms. However, its genome-wide effects in unicellular eukaryotic microalgae remain poorly characterized. This study developed a UV-C mutagenesis protocol in *Chlamydomonas reinhardtii* and evaluated its genomic and physiological impacts. Methods: Axenic cultures of *Chlamydomonas reinhardtii* (137c+) were exposed to UV-C (100–280 nm) for 12, 48, and 96 min. Viable colonies were analyzed by Random Amplification of Polymorphic DNA PCR (RAPD-PCR) to assess genetic variability, while chlorophyll content and the expression of stress-responsive genes were measured via spectrophotometry and Reverse Transcription quantitative Polymerase Chain Reaction (RT-qPCR), respectively. Results: UV-C treatment induced extensive genomic polymorphism with heterogeneous clustering patterns independent of exposure time, consistent with stochastic mutagenesis. Several mutants exhibited reduced chlorophyll content, indicating impaired photosynthetic efficiency. In contrast, one genotype (pop18) maintained wild-type chlorophyll levels despite marked genetic divergence, coupled with upregulation of antioxidant, DNA repair, and stress-response genes. Conclusions: Overall, UV-C irradiation represents an effective approach to generate non-directional genomic variability in *Chlamydomonas reinhardtii*, with evidence that random mutagenesis can drive functional reorganization of stress-response pathways, supporting its application in microalgal strain improvement.

## 1. Introduction

Microalgae are photosynthetic autotrophic microorganisms that have emerged as critical organisms for addressing global sustainability challenges [1]. These organisms are remarkable not only for their ability to perform oxygenic photosynthesis and split water into oxygen and protons, but also for their capacity to convert sunlight, carbon dioxide, and nutrients into valuable biomass through efficient light energy conversion [2,3]. The photosynthetic machinery of microalgae represents one of the most important biological processes on Earth, serving as the primary source of bioavailable energy and carbon for aquatic ecosystems while simultaneously offering promising biotechnological applications for biofuel production, carbon capture, and wastewater treatment [4,5].

The fundamental significance of microalgae photosynthesis lies in its versatility and efficiency. Unlike terrestrial plants, microalgae can rapidly accumulate biomass with carbon fixation efficiencies exceeding those of conventional crops, making them attractive candidates as “photosynthetic cell factories” for sustainable compound production [3]. Their rapid growth cycles, ability to thrive in diverse environmental conditions including non-arable land, and capacity to utilize wastewater as a nutrient source make them economically and ecologically advantageous [6]. Microalgae must survive in constantly changing environments and counteract abiotic and non-abiotic stresses [7]. Due to this extreme adaptability, the microalgal genome is very reactive and is able to vary in response to the selective pressures to which it is subjected [8]. It is possible to induce mutagenesis within microalgae using different technologies to simulate what happens in nature [9]. Strain isolation and mutagenesis of microalgae are efficient approaches to obtain specific phenotypes for high product yields [10]. *C. reinhardtii* is an oval-shaped eukaryotic unicellular alga about 10 μm long and 3 μm wide, with two long flagella in the anterior part that allow its movement [11]. *C. reinhardtii* is generally present as haploid vegetative cells with 17 chromosomes. There are two sexual types with opposite mating types (mt), denoted mt(+) and mt(−) [12]. Therefore, the algae can reproduce both sexually and asexually: in stressful conditions, such as lack of nutrients or overpopulation, it carries out sexual reproduction with the formation of a diploid zygote [13]. The zygote is not flagellated and will remain quiescent until more favorable environmental conditions [14]. Light influences the movements of the microalga and plays a fundamental role in the control of the sexual phase of the biological cycle; in fact, in the presence of light, the diploid zygote will germinate by carrying out meiosis, giving rise to four haploid cells (two mt(+) and two mt(−)) [15,16]. This occurs thanks to the regulatory action of phototropin, a receptor for blue light irradiation located in the flagella, whose signaling cascade is still unknown, and also responsible for chemotaxis movements in the presence of ammonium [17,18]. Today, *C. reinhardtii* is considered a model organism for microalgae studies; in fact, the knowledge of its genome and its metabolism is the most complete. Unlike terrestrial plants that can employ behavioral avoidance strategies, eukaryotic microalgae face persistent exposure to UV radiation as surface-dwelling photosynthetic organisms. Their survival depends entirely on the development of sophisticated molecular, physiological, and genetic defense mechanisms that have evolved over billions of years of fluctuating environmental conditions [19]. These adaptation mechanisms represent a remarkable example of evolution under intense selective pressure, where cells must either develop tolerance to UV damage or face cellular death.

Ultraviolet (UV) radiation represents one of the most significant environmental stressors affecting photosynthetic microorganisms globally [20]. The apparent increase in solar ultraviolet radiations on Earth’s surface, due to continuous depletion of the stratospheric ozone shield, has triggered serious ecological and biological consequences for microalgae and other life-forms [21]. The principal studies focused mainly on the effect of UV-A and UV-B radiations on different biological layer. It is known the exposure to UV radiation induces rapid and extensive upregulation of enzymatic antioxidant defenses that neutralize reactive oxygen species and prevent oxidative damage to cellular components [22]. In *C. reinhardtii*, oxidative stress induces increased expression of genes encoding enzymatic components of the ascorbate–glutathione system including peroxidase, superoxide dismutase, and dehydroascorbate reductase, alongside increased L-ascorbate accumulation. This coordinated upregulation of multiple components suggests sophisticated transcriptional regulation to ensure adequate antioxidant capacity. Similarly, in *Dunaliella salina*, enhanced antioxidant enzyme activities (SOD, CAT, and APX) were observed, indicating stress-induced activation of multiple antioxidant pathways [23,24]. However, UV-C radiation (100–280 nm) is the most energetic and harmful form of solar ultraviolet radiation. While Earth’s ozone layer and stratosphere primarily absorb most UV-C before it reaches the surface, understanding its mechanisms remains crucial for biology, sterilization applications, and studying extreme environments [25]. Usually, UV-C is highly effective at inducing severe DNA damage through two major pathways. The most significant lesions are cyclobutane pyrimidine dimers (CPDs) and 6-4 pyrimidine-pyrimidone photoproducts (6-4PPs), which distort the DNA helix [26]. The high resilience of microalgal communities in the face of UV-C stress is attributed to the activation of several integrated defense systems including photo/dark repair mechanisms, antioxidant systems, and biosynthesis of UV-photoprotectants such as mycosporine-like amino acids (MAAs), scytonemin, carotenoids, and polyamines [27]. As reported by Colina et al., low-intensity UV-C stress triggers protective cellular responses in *C. reinhardtii*. The algae respond through increased redox homeostasis, reactive oxygen species (ROS) scavenging, and protein damage repair mechanisms [28]. These physiological changes are accompanied by the upregulation of photosynthetic electron flux and carbon fixation processes. UV-C exposure also stimulates the production of secondary metabolites, including phenolic compounds and antioxidants, indicating an enhanced defense response [18]. Furthermore, *C. reinhardtii* shows adaptation through upregulation of genes involved in light-harvesting chlorophyll proteins, which help the algae to tolerate multiple abiotic stresses including UV-C radiation [12].

Understanding the genetic basis of UV adaptation in eukaryotic microalgae is particularly important given climate change, continued ozone depletion in some regions, and increasing UV exposure, especially in equatorial and high-altitude regions [10]. Additionally, biotechnological applications require development of UV-tolerant strains for outdoor cultivation and production of UV-protective bioactive compounds for pharmaceutical and cosmetic industries [25]. Considering, therefore, the potential applications of *C. reinhardtii*, this manuscript aimed to induce high selective pressure on microalgal cells through high mutagenic treatment with a UV-C radiation lamp to develop a genetic improvement program on algal colonies at different stages of growth [26]. The final objective is not only to develop a mutagenic protocol for differentiation and genetic selection but also to evaluate the possible effects of UV-C radiation on algal cultures that could be naturally affected as they are present in nature. For this reason, obtaining resistant genotypes could lead to their use for future applications of a different nature.

## 2. Materials and Methods

### 2.1. Cells Growth

We used the axenic *C. reinhardtii* 137c+ strain. The culture was grown in TAP medium under a light intensity of 120 mmol photons m^−2^s^−1^ in a 12 h:12 h light/dark cycle at 25 °C. Cultures were not supplied with an extra source of CO_2_ and were shaken by a mechanical agitator (g24 environmental incubator shaker, American Laboratory Trading-East Lyme, CT, USA) at 70 rpm.

One liter of TAP medium was prepared mixing: (i) 20 mL of 1 M Tris base; (ii) 1 mL of phosphate buffer (obtained dissolving 10.8 g of K_2_HPO_4_ and 5.6 g of KH_2_PO_4_ in 100 mL of distilled water); (iii) 10 mL of Solution A (obtained dissolving 20 g of NH_4_Cl, 5 g of MgSO_4_ ° 7 H_2_O and 2.5 g of CaCl_2_ ° 2H_2_O in 500 mL of distilled water); (iv) 1 mL of Hutner’s trace elements; (v) 1 mL of glacial acetic acid as described by Gorman et al. [15].

Cell growth was followed through a spectrophotometric analysis by measuring the optical density at 750 nm (SPECTROstar^®^ Nano, BMG Labtech, Ortenberg, Germany) and cell counts by light microscopy (Zeiss Axioplan, Oberndorf am Neckar, Germany) using a Burker chamber (BLAUBRAND, Wertheim Germany). All experiments were conducted in triplicate.

### 2.2. Mutagenesis

*C. reinhardtii* cells were subjected to a random mutagenesis process through the application of UV-C rays using an irradiation chamber equipped with a UV-C lamp (100–280 nm) (Helios Italquartz, Milan, Italy; model G15T8; characteristics: 15 W, 3.8 J m^−2^ at 1 m distance) [29].

Two different approaches were tested. At first, the algal cells were transferred onto Petri dishes and exposed to the action of UV-C directly on a solid medium, observing different timings based on data reported in the literature: overnight (about 12 h), 6 h, 3 h, 96 min, 48 min and 12 min [13]. The plates were then placed under 100 mmol·m^−2^·s^−1^ cold LED light (Philips TLD 30 W/55 lamp) at 25 °C to evaluate post-treatment growth. Subsequently, the exposure of the cultures in the liquid growth medium was evaluated.

In the second one, a smear of colonies was taken from a plate of *C. reinhardtii* 137c+, transferred into 50 mL of liquid medium (TAP) and stirred at 70 rpm (g24 environmental incubator shaker, American Laboratory Trading) under the same light and temperature conditions previously indicated. After two days, the mid-log phase was reached (approximately 10–12 × 10^6^ cells/mL). Once this concentration of cells was reached, 2 mL was taken and transferred into empty Petri dishes (60 mm diameter) to maximize exposure to UV-C of the thin cell films created. After exposure, observing the same time points, 10 μL aliquot was taken from each volume and transferred onto plates with solid medium (TAP) to encourage the growth of post-treatment colonies.

Subsequently, the isolated colonies obtained were transferred individually to other plates.

### 2.3. Chlorophyll Content

To analyze the possible effect of UV-C on photosynthetic activity, chlorophylls were evaluated before and after the treatment. Chlorophylls (Chl α and Chl b) were determined through a photometrical assay. Following Boussiba and Vonshak’s 1991 protocol [30], 100 µL of culture was centrifuged for 5 min at 18,000× *g*. The pellet was re-suspended into 100 µL of DMSO. The mixture was heated for 10 min at 70 °C. Extraction was repeated until a colorless pellet was obtained. For the supernatant, the optical density was determined at 649 nm/665 nm/480 nm. The concentration of chlorophylls (μg mL^−1^), was calculated according to Wellburn, 1994 [31].

Chl α = 12.19 A665 − 3.45 A649

Chl b = 21.99 A649 − 5.32 A665

### 2.4. Genetic Distances

Random Amplification of Polymorphic DNA PCR (RAPD-PRC) was conducted on the mutant strains and wild-type (WT) species to assess the genetic mutation induced by UV-C and to determine the genetic distance between them. Phire Plant Direct PCR Master Mix (Thermo Fisher Scientific, Waltham, MA, USA) was used for the DNA extraction and PCR reaction. Eight random primers (Table 1) were used, and 50 loci were highlighted. The reaction was checked with a 1.5% agarose gel (EMR010001, EuroClone S.p.a., Pero, Italy. Lot. 468.654). The gel was run using PowerPac 3000, (Bio-Rad, Hercules, CA, USA) at 80 V for 1 h. Population Genetic Analysis (POPGENE Version 1.32) software was used to analyze the differences. A dominant parameter was used to obtain the genetic distance (UPGMA method).

### 2.5. RNA Extraction, cDNA Synthesis, qPCR Assay

The RNA from WT and pop18 culture was extracted with NucleoZOL reagent (Macherey-Naghel, Düren Nordrhein-Westfalen, Germany) following the manufacturer’s recommendations. The RNA was quantified using a NanoDrop™1000 spectrophotometer (NanoDrop Technologies, Inc., Wilmington, DE, USA). RNA was retrotranscribed into cDNA using FIRE Script^®^ RT Cdna synthesis Mix (Solis Biodyne, Tartu, Estonia) and used at a final concentration of 10 ng. For the qPCR assay, Power SYBR Green PCR Master Mix (Applied Biosystems^®^, Waltham, MA, USA) was used in the following volumes: 5 _L of Power SYBR-Green PCR Master Mix (10X), 1.5 L of Forward Primer 10 mM, 1.5 _L of Reverse Primer 10 mM and 1 _L of the template.

The protocol was: (1) holding stage: 95 _C/10 min; (2) cycling stage: 40 cycles of 95 _C/15 s, 60 _C/1 min and 72 _C/1 min; (3) melt curve stage: 95 _C/15 s, 60 _C/1 min, 95_C/30 s and 60 _C/15 s. The 7500 Fast Real-Time PCR System (Applied Biosystems) and 7500 Software v2.3 (Applied Biosystems) were employed. The real-time PCR primers are reported in Table 2.

### 2.6. Statistical Analysis

The results were analyzed using Microsoft Excel 2016 and GraphPad Prism 8.0.2. All experiments were performed in biological triplicate, and data are reported as the mean ± standard error of the mean (SEM). A biological replicate refers to independent experimental samples derived from separate cultures grown and treated under the same conditions, allowing the assessment of biological variability among samples. In addition, for each biological replicate, three technical measurements were performed to ensure the accuracy and reproducibility of the analytical procedures.

Prior to statistical comparison, data distribution was evaluated for normality using the Shapiro–Wilk test. For comparisons among multiple UV-C-treated groups and the wild type, one-way analysis of variance (ANOVA) was applied, followed by Tukey’s multiple comparison post hoc test. When only two groups were compared, as in the RT-qPCR analysis between pop18 and WT, an unpaired Student’s *t*-test was used. Differences were considered statistically significant at *p* < 0.05.

For chlorophyll content, statistical comparisons were performed separately for chlorophyll a and chlorophyll b concentrations among the different colonies and the untreated wild type.

For RAPD-PCR analysis, amplified bands were scored as present or absent and converted into a binary matrix. Genetic distances were calculated using POPGENE v1.32, and cluster analysis was performed using the UPGMA method. The resulting dendrogram was used to evaluate genetic relationships among UV-C-treated colonies and the wild-type genotype.

For RT-qPCR data, Ct values were normalized against GAPDH, and relative gene expression was calculated using the 2^−ΔΔCt^ method, with WT used as the calibrator. Statistical analysis of gene expression was performed on ΔCt values, while fold-change values were used for graphical representation.

## 3. Results

### 3.1. Growth Dynamics of C. reinhardtii

**Figure 1:**
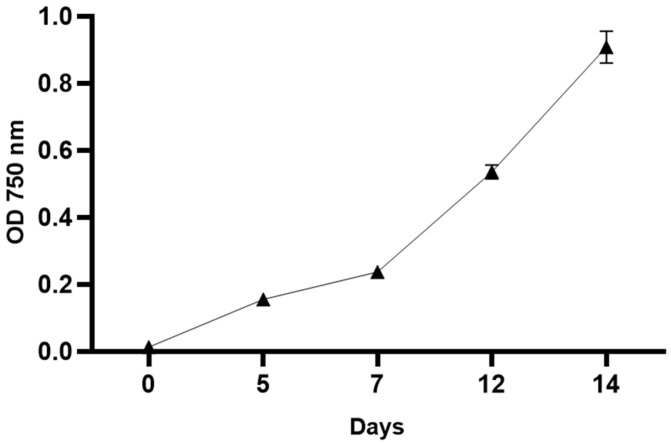
Growth curve of *C. reinhardtii* under standard culture conditions. The curve shows the typical growth pattern, including lag and exponential phases. Data are expressed as the mean ± standard error of the mean (SEM).

Under standard culture conditions, *C. reinhardtii* exhibited a typical growth pattern. As shown in Figure 1, the culture progressed through the expected growth phases and reached the exponential phase after approximately 7 days. The steady increase in optical density and cell number indicates that the culture was physiologically active and suitable for subsequent UV-C treatment.

### 3.2. Effect of UV-C Exposure on Cell Viability and Colony Recovery

Two different mutagenesis approaches were tested using the same UV-C irradiation system. When algal cells were exposed directly on solid medium, no viable colonies were recovered after treatment at any of the tested exposure times. In contrast, when UV-C irradiation was applied to liquid cultures, viable colonies were obtained following exposure times of 12, 48, and 96 min.

Cell viability was evaluated based on the ability of treated cells to form colonies on solid TAP medium after irradiation. In particular, only cells capable of resuming growth and generating visible colonies were considered viable.

The use of liquid cultures allowed the formation of a thin cell layer within the Petri dishes, ensuring homogeneous exposure to UV-C radiation while maintaining cell viability. After irradiation, aliquots were plated on solid TAP medium, and colony formation was observed for all three exposure times.

A total of 24 colonies were selected and isolated for further analysis, including eight colonies for each exposure time (12, 48, and 96 min) (Table 3).

### 3.3. Genetic Variability Induced by UV-C Radiation

RAPD-PCR analysis revealed the presence of polymorphic amplification patterns among the UV-C-treated colonies compared to the wild-type strain. A total of 50 loci were analyzed using eight random primers, and the resulting banding patterns were used to construct a binary matrix based on the presence or absence of bands.

**Figure 2:**
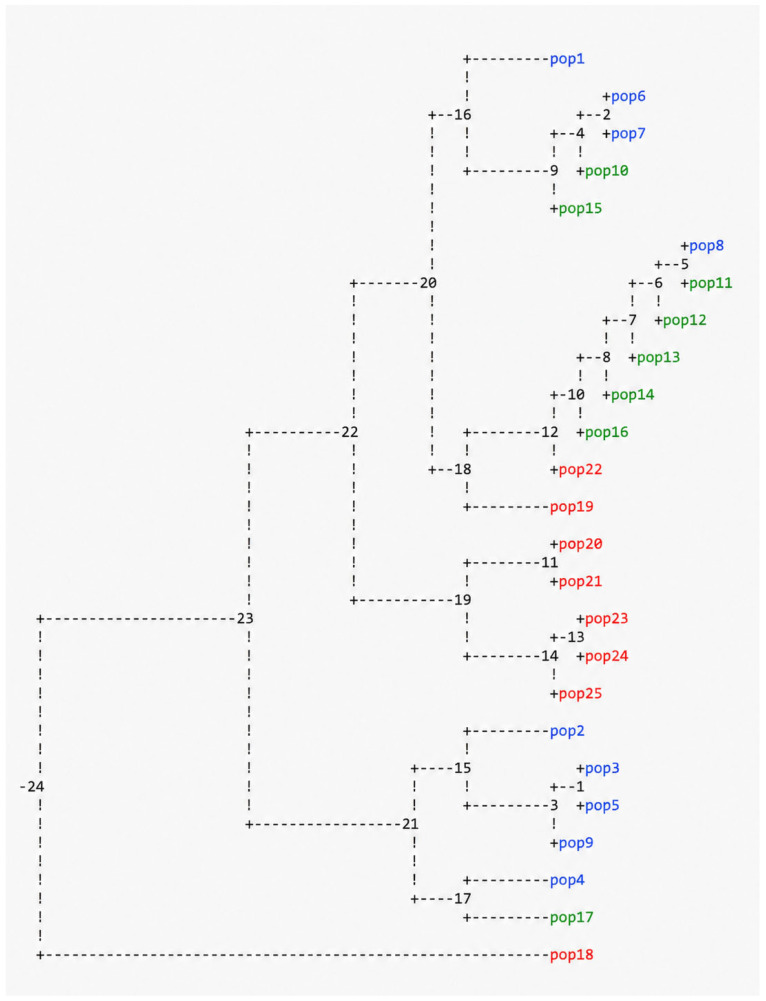
Dendrogram of genetic relationships among UV-C-treated colonies and wild-type *Chlamydomonas reinhardti*. Genetic distances were calculated based on RAPD-PCR banding patterns using a binary matrix (presence/absence of bands) and clustered using the UPGMA method. The wild type (WT) and colonies derived from different UV-C exposure times are included. Blue: samples treated for 12 min. Green: samples treated for 48 min. Red: samples treated for 96 min.

The dendrogram generated using the UPGMA method (Figure 2) showed a high level of genetic variability among the analyzed genotypes. Two main clusters were identified, along with a distinct genotype (pop18) that appeared clearly separated from all other samples.

Within the clusters, colonies derived from different exposure times were interspersed, and no clear grouping based on treatment duration was observed. Colonies exposed to the same UV-C duration were distributed across different branches of the dendrogram, indicating heterogeneous genetic responses to the treatment.

Some genotypes showed identical banding patterns, corresponding to a genetic distance of 0.000 and suggesting complete similarity at the analyzed loci. In contrast, other genotypes exhibited higher genetic distances, with pop18 showing the greatest divergence compared to both the wild type and the other treated colonies.

### 3.4. Effects of UV-C Treatment on Chlorophyll Content

**Figure 3:**
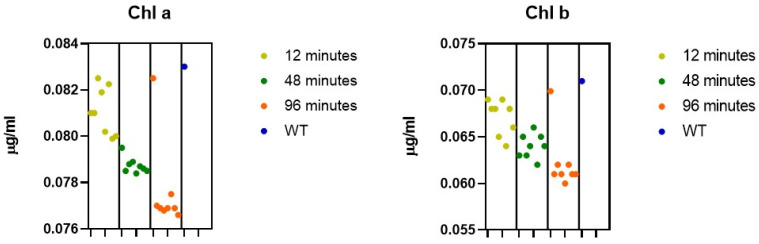
Chlorophyll content in UV-C-treated colonies of *C. reinhardtii* compared to the wild type (WT). Chlorophyll a (Chl a) and chlorophyll b (Chl b) concentrations were determined spectrophotometrically in untreated (WT) and UV-C-treated colonies obtained after 12, 48, and 96 min of exposure. Data are expressed as the mean ± standard error (SEM). Statistical significance was assessed as (*p* < 0.05).

Chlorophyll analysis revealed a general decrease in chlorophyll b content in UV-C-treated colonies compared to the wild type (Figure 3). This reduction was more pronounced in colonies exposed to longer irradiation times.

Chlorophyll a showed less variability across samples, whereas chlorophyll b exhibited a clearer trend of reduction following UV-C exposure.

Notably, the pop18 genotype displayed chlorophyll levels comparable to those of the wild type, despite belonging to the group exposed to the longest treatment duration (96 min). This behavior differed from that observed in the majority of the treated colonies, which showed a reduction in pigment content.

### 3.5. Transcriptional Response of Pop18 to UV-C Exposure

**Figure 4:**
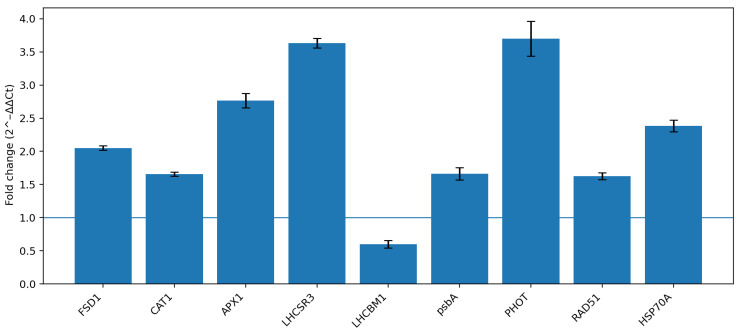
Relative gene expression analysis of pop18 compared to wild-type *C. reinhardtii*. Transcript levels of stress-responsive genes (*FSD1*, *CAT1*, *APX1*, *LHCBM*, *psbA*, *PHOT*, and *RAD51*) were quantified by RT-qPCR and normalized to *GAPDH* expression. Relative expression levels were calculated using the 2^−ΔΔCt^ method, with WT as the calibrator. Statistical significance was assessed as (*p* < 0.05).

RT-qPCR analysis revealed a significant modulation of genes associated with oxidative stress response, photosynthetic function, and DNA repair in the pop18 genotype compared to the wild type (WT) (Figure 4; Table 4).

Among the antioxidant-related genes, *FSD1*, *CAT1*, and *APX1* were all significantly upregulated. *FSD1* showed a clear increase in expression, while *CAT1* exhibited a moderate but consistent induction. *APX1* displayed the highest fold change among these genes.

For photosynthesis-related genes, *LHCBM* expression was significantly downregulated in pop18, whereas *psbA* showed a significant upregulation.

Regarding DNA repair-related genes, both *PHOT* and *RAD51* were significantly upregulated. *PHOT* exhibited one of the highest fold changes among all analyzed genes, while *RAD51* showed a lower but still statistically significant increase.

Overall, the transcriptional profile of pop18 is characterized by the simultaneous upregulation of genes involved in oxidative stress mitigation and DNA repair, together with a differential modulation of photosynthesis-related genes.

## 4. Discussion

The present study demonstrates that UV-C irradiation represents an effective mutagenic strategy to induce genomic variability in *C. reinhardtii*, while also revealing that the biological outcome of such mutagenesis is highly heterogeneous and not strictly dependent on exposure time. The experimental comparison between solid and liquid exposure conditions provides an initial methodological insight: the complete absence of viable colonies following irradiation on solid medium suggests that, under these conditions, UV-C exposure reaches a lethal threshold, likely due to the absence of shielding effects and the direct, prolonged interaction between radiation and cellular DNA. Conversely, the liquid-based exposure system appears to create a more permissive environment, enabling partial survival and subsequent selection of mutated colonies. This observation is particularly relevant for the development of controlled mutagenesis protocols, as it highlights the importance of physical exposure conditions in modulating mutagenic efficiency versus lethality. At the same time, this behavior closely reflects what occurs in natural aquatic environments, where microalgae are not exposed to UV radiation in a uniform or constant manner, but rather experience fluctuating irradiance levels modulated by water depth, turbidity, and self-shading within populations. In such contexts, sublethal UV exposure represents a key evolutionary pressure that promotes adaptation rather than extinction, allowing the selection of genotypes with enhanced tolerance mechanisms. Indeed, microalgae have evolved under variable UV regimes over geological timescales, developing integrated defense strategies that balance damage and survival, including DNA repair systems, antioxidant responses, and photoprotective compounds. The experimental system adopted in this study therefore mimics, to some extent, these natural selective dynamics, where only a fraction of the population survives and gives rise to genetically diversified lineages [11,24].

At the genomic level, RAPD-PCR analysis revealed extensive polymorphism among treated colonies, confirming that UV-C irradiation induces structural variation across the genome. Notably, the clustering analysis did not reflect the duration of exposure, indicating that mutagenesis does not proceed in a cumulative or dose-dependent linear manner, but rather follows a stochastic pattern. This behavior is consistent with the known mechanism of UV-C-induced DNA damage, which primarily involves the formation of cyclobutane pyrimidine dimers and 6-4 photoproducts occurring randomly along the DNA sequence. As a consequence, even relatively short exposure times may generate mutations comparable, in qualitative terms, to those induced by longer treatments. The presence of genetically identical profiles within certain groups further suggests that not all mutations are captured by RAPD markers, or that some genomic regions remain unaffected or selectively conserved.

In this context, the choice of RAPD-PCR as the analytical approach deserves specific consideration. The adoption of this technique was dictated by the intrinsic characteristics of the mutagenesis process itself. Since UV-C induces random and non-targeted genomic alterations, it is not possible to define, a priori, specific loci or candidate genes for targeted analysis. RAPD-PCR therefore represents a suitable exploratory tool, as it enables the detection of polymorphisms across multiple anonymous regions of the genome without requiring prior sequence information. This makes it particularly appropriate for first-level screening in non-directed mutagenesis studies. However, this approach also presents well-recognized limitations. RAPD markers are dominant and do not allow discrimination between allelic states, and their reproducibility may be influenced by PCR conditions and primer selection. Moreover, the technique provides only a partial and low-resolution representation of genomic variability, capturing a subset of mutations without enabling their precise localization or functional annotation. Consequently, while RAPD analysis effectively highlights patterns of genetic divergence, it cannot resolve the molecular basis of the observed phenotypes. Despite these constraints, in the framework of this study, RAPD-PCR proved adequate to demonstrate the extent and randomness of UV-C-induced variation and to identify highly divergent genotypes such as pop18, which represent valuable targets for further high-resolution genomic analyses.

Within this context of widespread but random genomic variation, the identification of the pop18 genotype as the most divergent individual is particularly significant. Its isolated position in the dendrogram indicates that UV-C exposure has generated a markedly distinct genomic configuration. However, this divergence does not correspond to a detrimental phenotype; on the contrary, pop18 maintains physiological parameters comparable to the wild type, particularly with respect to chlorophyll content. This apparent decoupling between genetic divergence and physiological impairment suggests that UV-C-induced mutagenesis can give rise not only to deleterious effects, but also to functional reorganization of cellular processes.

The analysis of chlorophyll content supports this interpretation. Most UV-C-treated colonies exhibited a reduction in chlorophyll b levels, consistent with the well-established sensitivity of the photosynthetic apparatus to oxidative stress. UV-C radiation is known to promote the formation of reactive oxygen species, which directly damage chloroplast structures and pigment–protein complexes. The observed decrease in chlorophyll b, in particular, reflects disruption of light-harvesting complexes, leading to reduced photosynthetic efficiency. In contrast, the maintenance of chlorophyll levels in pop18 indicates the presence of protective or compensatory mechanisms that mitigate oxidative damage. A plausible explanation for this observation is that UV-C exposure may have selected for mutations enhancing the activation or efficiency of endogenous photoprotective and antioxidant systems. In microalgae, oxidative stress is known to trigger the upregulation of enzymatic antioxidants and the biosynthesis of protective pigments such as carotenoids, which can absorb excess radiation and quench reactive oxygen species [17]. Additionally, UV-induced stress can stimulate metabolic reprogramming leading to increased pigment production or improved redox homeostasis, thereby preserving chlorophyll integrity under adverse conditions [32].

It is therefore reasonable to hypothesize that pop18 may have acquired mutations affecting regulatory pathways controlling these processes, resulting in a more efficient balance between light absorption [28] and energy dissipation, ultimately maintaining photosynthetic functionality despite UV-induced stress [30]. 

This hypothesis is strongly supported by the transcriptional profiling data.

The transcriptional profile observed in the pop18 genotype provides a mechanistic framework to interpret its distinctive physiological behavior and its ability to maintain chlorophyll content despite strong UV-C exposure. The coordinated modulation of genes involved in oxidative stress response, photosynthetic regulation, and DNA repair suggests that pop18 does not simply tolerate stress passively, but actively reorganizes key cellular pathways.

The upregulation of *FSD1*, *CAT1*, and *APX1* clearly indicates the activation of a multi-level antioxidant defense system. These enzymes operate in a sequential and functionally interconnected manner. *FSD1* catalyzes the dismutation of superoxide radicals into hydrogen peroxide, which is subsequently detoxified by *CAT1* and *APX1* through catalase-dependent and ascorbate-dependent pathways, respectively. The strong induction of *APX1*, in particular, suggests a prominent role of the ascorbate–glutathione cycle in maintaining redox homeostasis. This is especially relevant in photosynthetic organisms, where chloroplasts represent a major source of ROS under stress conditions. The simultaneous upregulation of these genes indicates that pop18 likely possesses an enhanced capacity to rapidly neutralize ROS, thereby limiting oxidative damage to membranes, proteins, and pigments.

This enhanced antioxidant capacity provides a plausible mechanistic explanation for the preservation of chlorophyll content observed in pop18. Under UV-C stress, ROS accumulation typically leads to degradation of chlorophylls and damage to the photosynthetic apparatus. However, in pop18, the efficient scavenging of ROS may prevent or significantly reduce such damage, maintaining pigment stability and overall photosynthetic functionality.

The transcriptional modulation of photosynthesis-related genes further supports this interpretation. The downregulation of *LHCBM* suggests a reduction in the size or activity of the light-harvesting antenna complexes. This adjustment can be interpreted as a protective strategy aimed at limiting the absorption of excess light energy, which under stress conditions could otherwise exacerbate ROS production. By reducing excitation pressure on the photosystems, the cell minimizes the risk of photoinhibition and oxidative damage.

Conversely, the upregulation of *psbA*, encoding the D1 protein of photosystem II (PSII), indicates an active maintenance of PSII repair mechanisms. The D1 protein is one of the primary targets of photodamage and undergoes rapid turnover under stress conditions. Increased *psbA* expression suggests that pop18 enhances its capacity to replace damaged D1 protein, thereby sustaining PSII functionality. The combination of reduced light harvesting (via *LHCBM* downregulation) and enhanced PSII repair (via *psbA* upregulation) reflects a coordinated strategy to stabilize photosynthetic performance under UV-induced stress.

In addition to oxidative and photosynthetic responses, the transcriptional activation of *PHOT* and *RAD51* highlights the importance of DNA repair processes in the adaptive response of pop18. *PHOT* is associated with light-dependent DNA repair mechanisms, particularly the reversal of UV-induced lesions such as pyrimidine dimers. Its strong upregulation suggests an increased efficiency in direct photoreactivation pathways, enabling rapid restoration of DNA integrity.

*RAD51*, on the other hand, plays a central role in homologous recombination, a high-fidelity repair mechanism for double-strand breaks and complex DNA lesions. The upregulation of *RAD51* indicates that pop18 is also capable of activating recombination-based repair pathways, which are essential when damage exceeds the capacity of direct repair systems. The simultaneous activation of both photoreactivation and homologous recombination suggests that pop18 has developed a robust and versatile DNA repair network.

Taken together, these transcriptional changes delineate an integrated stress-response phenotype in which antioxidant defense, photosynthetic regulation, and DNA repair are tightly coordinated. This integrated response likely underlies the ability of pop18 to maintain physiological stability despite extensive genomic variation induced by UV-C radiation.

Importantly, these results reinforce the concept that random mutagenesis can generate not only genomic diversity but also emergent regulatory configurations that enhance stress tolerance. In this case, the combination of enhanced ROS detoxification, controlled energy input into the photosystems, sustained PSII repair, and efficient DNA damage resolution appears sufficient to counterbalance the detrimental effects of UV-C exposure. This suggests that the adaptive potential of microalgae under mutagenic stress is strongly dependent on the reorganization of regulatory networks rather than on the extent of genomic change alone.

## 5. Conclusions

This study establishes an effective and reproducible UV-C-based mutagenesis protocol for *C. reinhardtii*, demonstrating that controlled irradiation of liquid cultures enables the recovery of viable colonies with substantial genomic diversification. Unlike conventional assumptions that mutagenic effects scale with exposure time, our results show that UV-C-induced genomic variation is largely stochastic and not directly correlated with treatment duration, providing experimental evidence of the non-directional nature of UV-C mutagenesis in microalgae. A key finding of this work is that genomic variability generated through random mutagenesis is not merely structural, but can be associated with the emergence of functionally coherent phenotypes. By integrating RAPD-based genomic profiling with targeted gene expression analysis, we demonstrate that specific genotypes, such as pop18, exhibit a coordinated reprogramming of stress-response pathways, including antioxidant defense, photosynthetic regulation, and DNA repair. This highlights that random mutagenesis can give rise to adaptive regulatory configurations rather than exclusively deleterious effects. Importantly, our data suggest that relatively limited and non-targeted genomic modifications are sufficient to induce significant functional changes at the cellular level. This finding contributes to a better understanding of how microalgal systems can translate stochastic genomic perturbations into structured physiological responses, providing insight into the mechanisms underlying stress adaptation in photosynthetic microorganisms.

From an applied perspective, this study demonstrates that UV-C mutagenesis is not only a tool for generating genetic diversity, but also a strategy for selecting phenotypes with enhanced tolerance to oxidative and radiation stress. The identification of genotypes capable of maintaining photosynthetic functionality under extreme conditions underscores the potential of this approach for the development of robust microalgal strains for biotechnological applications, particularly in outdoor cultivation systems exposed to environmental stressors.

Overall, this work advances current knowledge by linking random UV-C-induced genomic variation to functional cellular outcomes, providing both a methodological framework for mutagenesis in microalgae and new insights into the relationship between genomic plasticity and stress adaptation.

## Figures and Tables

**Table 1:** Primers used in RAPD-PCR.

Primer	% GC
5′-GGTCGCAGCT-3′	60
5′-CGGACCGCGT-3′	80
5′-AGACGTACTC-3′	50
5′-AGTCATGCCAC-3′	54
5′-GGGTAACTGCC-3′	63
5′-TTCGGCACGGG-3′	72
5′CTTTTCGCTGGGAGA-3′	53
5′CCGTCGGCCAATTGG-3′	66

**Table 2:** Primers used in real-time PCR.

Gene	Sequence Primer (5′→3′)	Amplicon (bp)	Tm	GC%	Notes
*GADPH*	Forward: AGAAGGCTGGGGCTCATTTG	148	62	55	
	Reverse: AGGGGCCATCCACAGTCTTC		64	60	
*FSD1*	Forward:ACTACATTGACGTGCAGAACCGCC	112	60	54	Nuclear gene encoding chloroplast Fe-SOD1.
	Reverse: ACCAATTACTTGGTGGCAGCGGC		60	56	
*CAT1*	Forward: ATGGTTCACGCCTTCAAGCCCAAC	104	60	54	Catalase 1.
	Reverse: AACGTCAGCATGTGGCATGACTCG		60	54	
*APX1*	Forward: AGATTGACCATGGCGCCAACAAGG	110	60	54	Ascorbate peroxidase 1.
	Reverse: TGATGCCATTTGGAACAGGTCGGC		60	54	
*LHCBM*	Forward: AGCTTGAGTTGTTTGGGAGCGGTG	109	60	54	Major light-harvesting complex II protein m1 target.
	Reverse: AAACGTTGACAAGTCCCAGCGCAG		61	54	
*psbA*	Forward: AGCTGCTCACGGTTACTTTGGTCG	99	59	54	Chloroplast-encoded PSII D1 gene.
	Reverse: CCGATTACCGGCCAAGCAGCTAAG		60	58	
*PHOT*	Forward: ATGAAGCTGATGCACTTCCGTCGC	119	60	54	Phototropin/putative blue-light receptor.
	Reverse: AACTTGTCCAGCGTCTTCATCGCG		60	54	
*RAD51*	Forward: GGTGCAAGGAGCTCGACACCATTC	112	60.00	58	DNA repair protein RAD51.
	Reverse: ACACACAGTGTGTGGCACAGCTG		59	56	

**Table 3:** Identification codes of the samples present in the dendrogram. Blue: samples treated for 12 min. Green: samples treated for 48 min. Red: samples treated for 96 min.

Sample	Code in the Dendrogram	Sample	Code in the Dendrogram
Wild type	Pop 1	Colony 5—48 min	Pop 14
Colony 1—12 min	Pop 2	Colony 6—48 min	Pop 15
Colony 2—12 min	Pop 3	Colony 7—48 min	Pop 16
Colony 3—12 min	Pop 4	Colony 8—48 min	Pop 17
Colony 4—12 min	Pop 5	Colony 1—96 min	Pop 18
Colony 5—12 min	Pop 6	Colony 2—96 min	Pop 19
Colony 6—12 min	Pop 7	Colony 3—96 min	Pop 20
Colony 7—12 min	Pop 8	Colony 4—96 min	Pop 21
Colony 8—12 min	Pop 9	Colony 5—96 min	Pop 22
Colony 1—48 min	Pop 10	Colony 6—96 min	Pop 23
Colony 2—48 min	Pop 11	Colony 7—96 min	Pop 24
Colony 3—48 min	Pop 12	Colony 8—96 min	Pop 25
Colony 4—48 min	Pop 13		

**Table 4:** Relative expression levels of genes reported as fold change, log2 fold change (log2FC), standard error of the mean (SEM), and *p*-value.

Gene	Fold Change	log2FC	SEM	*p*-Value
*FSD1*	2.051	1.037	0.034	0.0004
*CAT1*	1.657	0.728	0.031	0.0009
*APX1*	2.767	1.468	0.109	0.0006
*LHCSR3*	3.634	1.862	0.072	0.0000
*LHCBM1*	0.598	−0.742	0.056	0.0177
*psbA*	1.661	0.732	0.094	0.0054
*PHOT*	3.702	1.888	0.264	0.0002
*RAD51*	1.625	0.700	0.050	0.0005
*HSP70A*	2.384	1.253	0.088	0.0006

## Data Availability

The original contributions presented in this study are included in the article. Further inquiries can be directed to the corresponding author.

## References

[1] Jareonsin S., Mahanil K., Duangjan K., Srinuanpan S., Pekkoh J., Ishii M., Pumas C. (2023). Dark Mode of Microalga—A Sustainable and Economical Solution for Microalgal Biofuel Production and Waste Treatment. Bioresour. Technol. Rep..

[2] Li X., Stegen J.C., Yu Y., Huang J. (2023). Coordination and Divergence in Community Assembly Processes across Co-Occurring Microbial Groups Separated by Cell Size. Front. Microbiol..

[3] Lee S.M., Ryu C.M. (2021). Algae as New Kids in the Beneficial Plant Microbiome. Front. Plant Sci..

[4] Cao K., Cui Y., Sun F., Zhang H., Fan J., Ge B., Cao Y., Wang X., Zhu X., Wei Z. (2023). Metabolic Engineering and Synthetic Biology Strategies for Producing High-Value Natural Pigments in Microalgae. Biotechnol. Adv..

[5] Kholssi R., Lougraimzi H., Moreno-Garrido I. (2023). Effects of Global Environmental Change on Microalgal Photosynthesis, Growth and Their Distribution. Mar. Environ. Res..

[6] Stonik V.A., Stonik I.V. (2023). Carbohydrate-Containing Low Molecular Weight Metabolites of Microalgae. Mar. Drugs.

[7] Cheng J., Lu H., Huang Y., Li K., Huang R., Zhou J., Cen K. (2016). Enhancing Growth Rate and Lipid Yield of Chlorella with Nuclear Irradiation under High Salt and CO_2_ Stress. Bioresour. Technol..

[8] Radice R.P., Fiorentino R., De Luca M., Limongi A.R., Viviano E., Bermano G., Martelli G. (2021). An Innovative Protocol to Select the Best Growth Phase for Astaxanthin Biosynthesis in *H. Pluvialis*. Biotechnol. Rep..

[9] Jeong B., Jang J., Jin E. (2023). Genome Engineering via Gene Editing Technologies in Microalgae. Bioresour. Technol..

[10] Sun H., Wu T., Chen S.H.Y., Ren Y., Yang S., Huang J., Mou H., Chen F. (2021). Powerful Tools for Productivity Improvements in Microalgal Production. Renew. Sust. Energy Rev..

[11] Masi A., Leonelli F., Scognamiglio V., Gasperuzzo G., Antonacci A., Terzidis M.A. (2023). *Chlamydomonas reinhardtii*: A Factory of Nutraceutical and Food Supplements for Human Health. Molecules.

[12] Huang K., Kunkel T., Beck C.F. (2004). Localization of the Blue-Light Receptor Phototropin to the Flagella of the Green Alga *Chlamydomonas reinhardtii*. Mol. Biol. Cell.

[13] Rochaix J.D., Maloy S., Hughes K. (2013). *Chlamydomonas reinhardtii*. Brenner’s Encyclopedia of Genetics.

[14] Pröschold T., Harris E.H., Coleman A.W. (2005). Portrait of a Species: *Chlamydomonas reinhardtii*. Genetics.

[15] Gorman D.S., Levine R.P. (1965). Cytochrome f and Plastocyanin: Their Sequence in the Photosynthetic Electron Transport Chain of Chlamydomonas Reinhardi. Proc. Natl. Acad. Sci. USA.

[16] Tian L., Zhang Z., Wang Z., Zhang P., Xiong C., Kuang Y., Peng X., Yu M., Qian Y. (2023). Compositional Variations in Algal Organic Matter during Distinct Growth Phases in Karst Water. Front. Environ. Sci..

[17] Sun B., Guo X., Fan C., Chen Y., Wang J., Hu Z. (2018). Newly Identified Essential Amino Acids Affecting Chlorella Ellipsoidea DGAT1 Function Revealed by Site-Directed Mutagenesis. Int. J. Mol. Sci..

[18] Liu S., Zhao Y., Liu L., Ao X., Ma L., Wu M., Ma F. (2015). Improving Cell Growth and Lipid Accumulation in Green Microalgae Chlorella Sp. via UV Irradiation. Appl. Biochem. Biotechnol..

[19] Sneeden J.L., Loeb L.A. (2003). Random Oligonucleotide Mutagenesis. Directed Evolution Library Creation.

[20] Katerova Z., Todorova D. (2012). Influence of Ultraviolet Radiation on Plant Secondary Metabolite Production. Genet. Plant Physiol..

[21] Vanhaelewyn L., Van Der Straeten D., De Coninck B., Vandenbussche F. (2020). Ultraviolet Radiation From a Plant Perspective: The Plant-Microorganism Context. Front. Plant Sci..

[22] Umar S.A., Tasduq S.A. (2022). Ozone Layer Depletion and Emerging Public Health Concerns—An Update on Epidemiological Perspective of the Ambivalent Effects of Ultraviolet Radiation Exposure. Front. Oncol..

[23] Zhang J., Liu L., Ren Y., Chen F. (2019). Characterization of Exopolysaccharides Produced by Microalgae with Antitumor Activity on Human Colon Cancer Cells. Int. J. Biol. Macromol..

[24] Lagoda P.J.L. (2012). Effects of Radiation on Living Cells and Plants. Plant Mutation Breeding and Biotechnology.

[25] Abbasi B.H., Khan T., Khurshid R., Nadeem M., Drouet S., Hano C. (2021). UV-C Mediated Accumulation of Pharmacologically Significant Phytochemicals under Light Regimes in in Vitro Culture of *Fagonia Indica* (L.). Sci. Rep..

[26] Pradana Y.S., Nugraha B.A., Sari W., Sadewo B.R., Dewayanto N. (2023). Application of Low-Dose UV-C for Microalgae *Spirulina* Sp. Sterilization. AIP Conf. Proc..

[27] Carino J.D., Vital P.G. (2023). Characterization of Isolated UV-C-Irradiated Mutants of Microalga Chlorella Vulgaris for Future Biofuel Application. Environ. Dev. Sustain..

[28] Colina F., Carbó M., Meijón M., Cañal M.J., Valledor L. (2020). Low UV-C Stress Modulates *Chlamydomonas reinhardtii* Biomass Composition and Oxidative Stress Response through Proteomic and Metabolomic Changes Involving Novel Signalers and Effectors. Biotechnol. Biofuels.

[29] Castronuovo D., Tataranni G., Lovelli S., Candido V., Sofo A., Scopa A. (2014). UV-C Irradiation Effects on Young Tomato Plants: Preliminary Results. Pak. J. Bot..

[30] Boussiba S., Vonshak A. (1991). Astaxanthin Accumulation in the Green Alga *Haematococcus Pluvialis*. Plant Cell Physiol..

[31] Wellburn A.R. (1994). The Spectral Determination of Chlorophylls a and b, as Well as Total Carotenoids, Using Various Solvents with Spectrophotometers of Different Resolution. J. Plant Physiol..

[32] Lorena Almaraz-Delgado A., Flores-Uribe J., Hugo Pérez-España V., Salgado-Manjarrez E., Agustín Badillo-Corona J. (2014). Production of Therapeutic Proteins in the Chloroplast of *Chlamydomonas reinhardtii*. AMB Express.

